# Atypical Polycystic Kidney Disease as defined by Imaging

**DOI:** 10.1038/s41598-022-24104-w

**Published:** 2023-02-20

**Authors:** Ioan-Andrei Iliuta, Aung Zaw Win, Matthew B. Lanktree, Seung Heyck Lee, Marina Pourafkari, Fatemeh Nasri, Elsa Guiard, Amirreza Haghighi, Ning He, Alistair Ingram, Crystal Quist, David Hillier, Korosh Khalili, York Pei

**Affiliations:** 1 Division of Nephrology, Department of Medicine, University Health Network and University of Toronto, 8N838, 585 University Avenue, Toronto, ON M5G 2N2 Canada; 2 Department of Medical Imaging, University Health Network and University of Toronto, Toronto, ON Canada; 3 Division of Nephrology, Department of Medicine, St. Joseph’s Healthcare and McMaster University, Hamilton, ON Canada; 4 CIHR SPOR Can-SOLVE Network, Vancouver, Canada

**Keywords:** Diseases, Health care, Nephrology, Risk factors

## Abstract

Using age- and height-adjusted total kidney volume, the Mayo Clinic Imaging Classification provides a validated approach to assess the risk of chronic kidney disease (CKD) progression in autosomal dominant polycystic kidney disease (ADPKD), but requires excluding patients with atypical imaging patterns, whose clinical characteristics have been poorly defined. We report an analysis of the prevalence, clinical and genetic characteristics of patients with atypical polycystic kidney disease by imaging. Patients from the extended Toronto Genetic Epidemiology Study of Polycystic Kidney Disease recruited between 2016 and 2018 completed a standardized clinical questionnaire, kidney function assessment, genetic testing, and kidney imaging by magnetic resonance or computed tomography. We compared the prevalence, clinical features, genetics, and renal prognosis of atypical versus typical polycystic kidney disease by imaging. Forty-six of the 523 (8.8%) patients displayed atypical polycystic kidney disease by imaging; they were older (55 vs. 43 years; *P* < 0.001), and less likely to have a family history of ADPKD (26.1% vs. 74.6%; *P* < 0.001), a detectable *PKD1* or *PKD2* mutation (9.2% vs. 80.4%; *P* < 0.001), or progression to CKD stage 3 or stage 5 (*P* < 0.001). Patients with atypical polycystic kidney disease by imaging represent a distinct prognostic group with a low likelihood of progression to CKD.

## Introduction

Autosomal dominant polycystic kidney disease (ADPKD) is the most common inherited kidney disorder with a prevalence of at least 1/1000, and is an important cause of end-stage kidney disease (ESKD)^1–3^. Progressive cyst expansion distorts the kidney architecture and ultimately leads to ESKD in a large proportion of patients^4^. With the recent approval of Tolvaptan as the first disease-modifier drug for ADPKD^5,6^, identifying high-risk patients who may benefit from this treatment is a clinical priority^7,8^. The Consortium for Radiologic Imaging Studies of Polycystic Kidney Disease has shown that total kidney volume (TKV) expands quasi-exponentially during adult life at ~ 5% per year and is a sensitive marker for predicting chronic kidney disease (CKD) progression in ADPKD^9^.

Using age- and height-adjusted TKV determined by magnetic resonance imaging (MRI), the Mayo Clinic Imaging Classification (MCIC) provides a validated approach for CKD risk stratification^10,11^, for enrichment of high-risk patients in clinical trials^11,12^, and is now commonly used in clinical practice^13^. However, it requires visual inspection of MR images to exclude cases with atypical imaging patterns (class 2) which were present in 8.8% (52/590) of patients from the Mayo Clinic derivation cohort but excluded from subsequent analyses^10^. The typical imaging (class 1) pattern for the Mayo Clinic Imaging Classification is defined as bilateral and diffuse cyst distribution, where all cysts similarly contribute to TKV. By contrast, atypical polycystic kidney disease is defined by one of the following imaging patterns: (i) unilateral, (ii) asymmetric, (iii) segmental, (iv) lopsided, or bilateral cystic disease with (v) unilateral or (vi) bilateral kidney atrophy (Table 1)^10^. Patients with atypical polycystic kidney disease by imaging represent a distinct clinical population which has not been well characterized. Here, we report a systematic study to define the prevalence and clinical characteristics of patients with atypical polycystic kidney by imaging.

**Table 1:** Toronto updated classification of cystic kidney imaging patterns^a^.

Class	Subclass	Subclass name	Description
1. Typical	1A, 1B	Mild	Bilateral and diffuse distribution, with mild, moderate, or severe replacement of kidney tissue by cysts, where all cysts contribute similarly to total kidney volume
	1C, 1D, 1E	Severe	
2. Atypical	2A	Unilateral	Diffuse cystic involvement of one kidney causing marked renal enlargement with a normal contralateral kidney defined by a normal kidney volume (< 275 mL in men; < 244 mL in women) and having less than 3 cysts
		Segmental	Cystic disease involving only one pole of one or both kidneys and sparing the remaining renal tissue
		Asymmetric	Diffuse cystic involvement of one kidney causing marked enlargement with mild segmental or minimal diffuse involvement of the contralateral kidney defined by a small number of cysts (> 2 but < 10) and volume < 30% of TKV
		Lopsided	Bilateral distribution of renal cysts with mild replacement of kidney tissue with atypical cysts where 2–5 cysts account for ≥ 50% total kidney volume
		Segmental sparing^b^	Bilateral and diffuse distribution with sparing of one pole of one or both kidneys
		Mild lopsided^b^	Bilateral distribution of renal cysts with mild replacement of kidney tissue with atypical cysts where 2–5 cysts account for 15–49% total kidney volume
	2B	Bilateral presentation with acquired unilateral atrophy	Diffuse cystic involvement of one kidney causing moderate to severe renal enlargement with contralateral acquired atrophy
		Bilateral presentation with acquired bilateral atrophy	No significant enlargement of the kidneys, defined by an average length < 14.5 cm, and replacement of kidney tissue by cysts with atrophy of the parenchyma

^a^Adapted from the Mayo Clinic Imaging Classification; ^b^two new patterns added in the current study.

## Results

### Prevalence of atypical imaging patterns in patients with polycystic kidney disease

**Figure 1:**
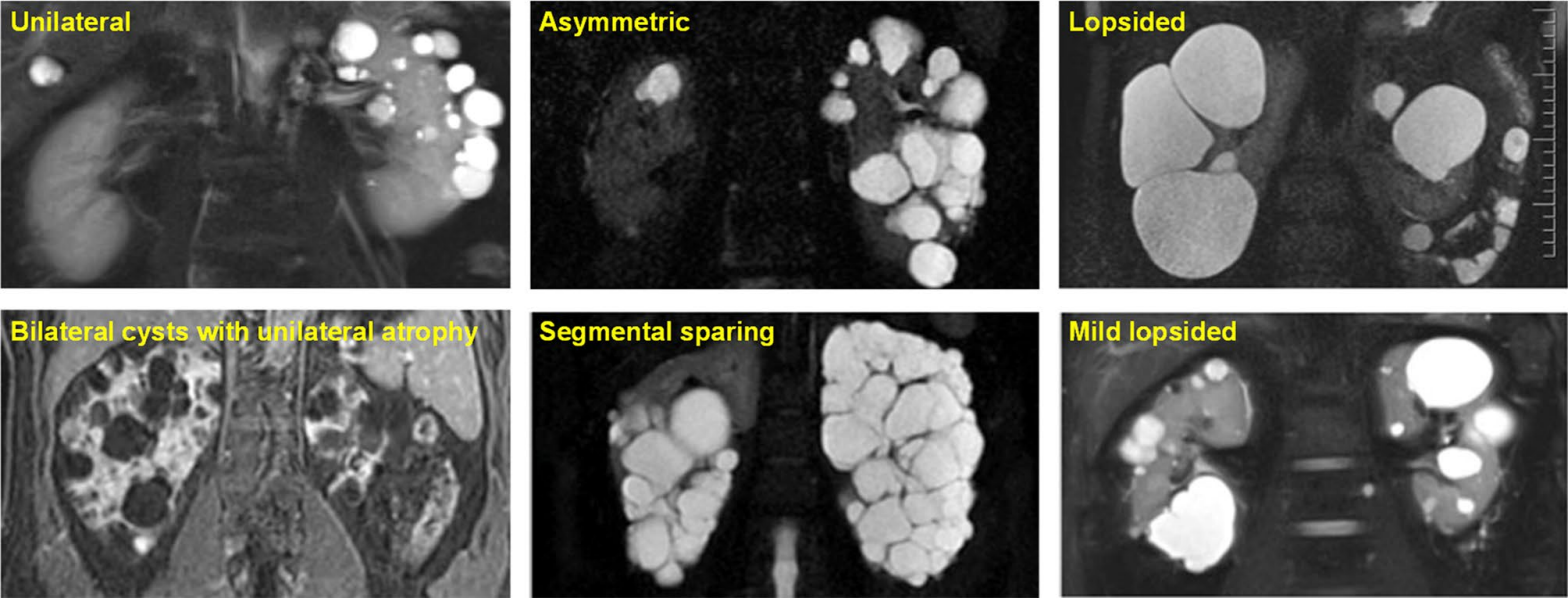
Illustrations of atypical imaging patterns. These include: unilateral (diffuse cystic involvement of only one kidney); asymmetric (diffuse cystic involvement of one kidney with mild involvement of the contralateral kidney); lopsided (bilateral distribution of cysts with mild replacement of kidney tissue with atypical cysts where 2–5 cysts account for ≥ 50% of total kidney volume); bilateral presentation with acquired unilateral atrophy (diffuse cystic involvement of one kidney with contralateral acquired atrophy); segmental sparing (bilateral and diffuse distribution with sparing of one kidney pole); mild lopsided (same definition as lopsided, but with the larger cysts accounting for 15–49% of total kidney volume).

From this cohort of 543 patients who presented with a clinical diagnosis suggestive of ADPKD, 20 cases were excluded because of incomplete clinical data (n = 7); non-ADPKD diagnoses (n = 8) including simple cysts (n = 4), peri-pelvic cysts (n = 2), congenital anomalies of the kidney and urinary tract (n = 1), and cystic disease related to a *COL4A1* mutation (n = 1); complex diagnoses of ADPKD with a second kidney disease (n = 2); and no *PKD1* and *PKD2* mutation results available (n = 3) (Supplementary Fig. S1). After reviewing their MRI, we found a prevalence of atypical kidney imaging patterns in 8.8% (46/523) of the study patients (Table 1): 1 unilateral, 10 asymmetric, 9 lopsided, 1 bilateral presentation with acquired unilateral atrophy, 5 segmental sparing, and 20 mild lopsided (see Fig. 1 for illustrative examples).

### Clinical characteristics of patients with atypical polycystic kidney disease by imaging

**Figure 2:**
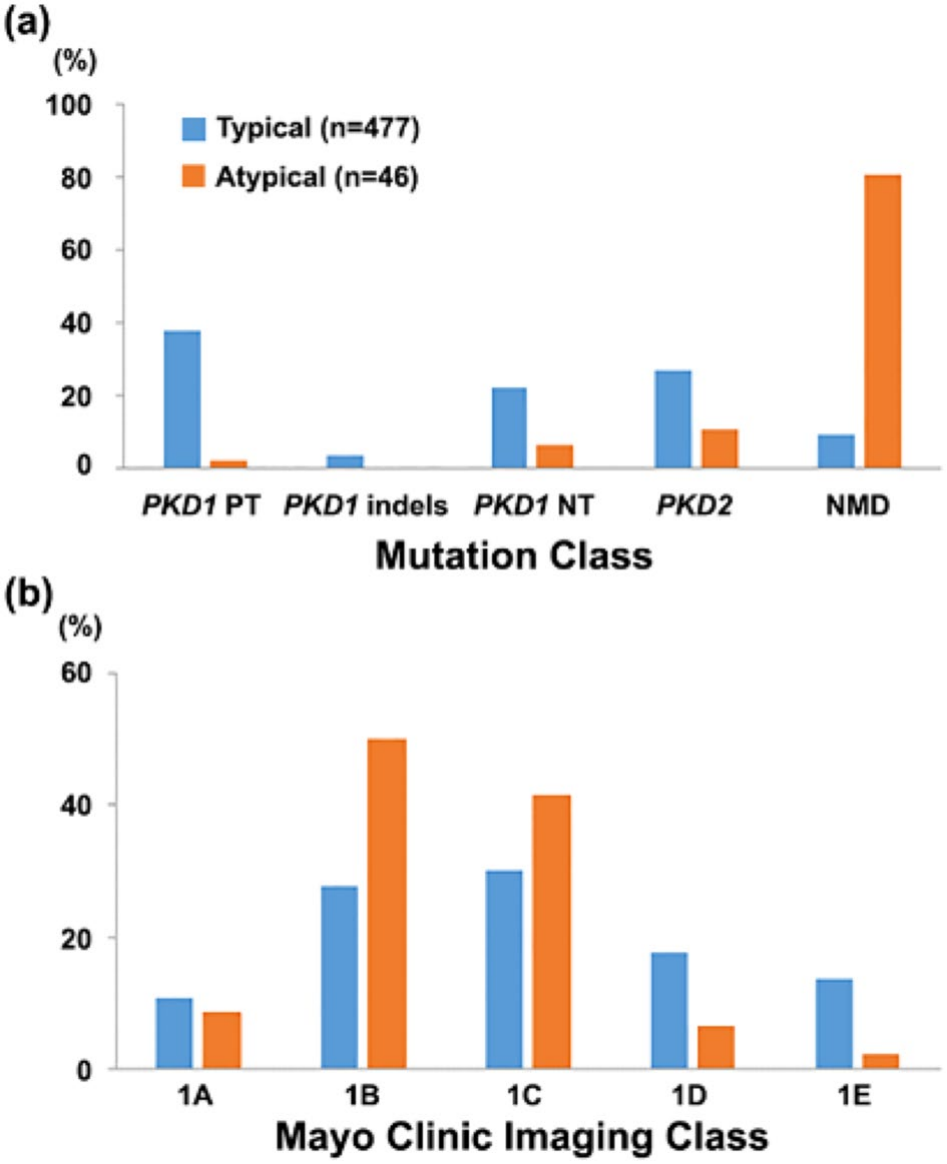
(**a**) Distribution of mutation classes in patients with typical and atypical polycystic kidney disease by imaging; and (**b**) Mayo Clinic Imaging Classification of all patients, including the misclassification of potentially unrecognized patients with atypical imaging patterns. NMD: no mutation detected; NT: non-truncating; PT: protein-truncating.

**Figure 3:**
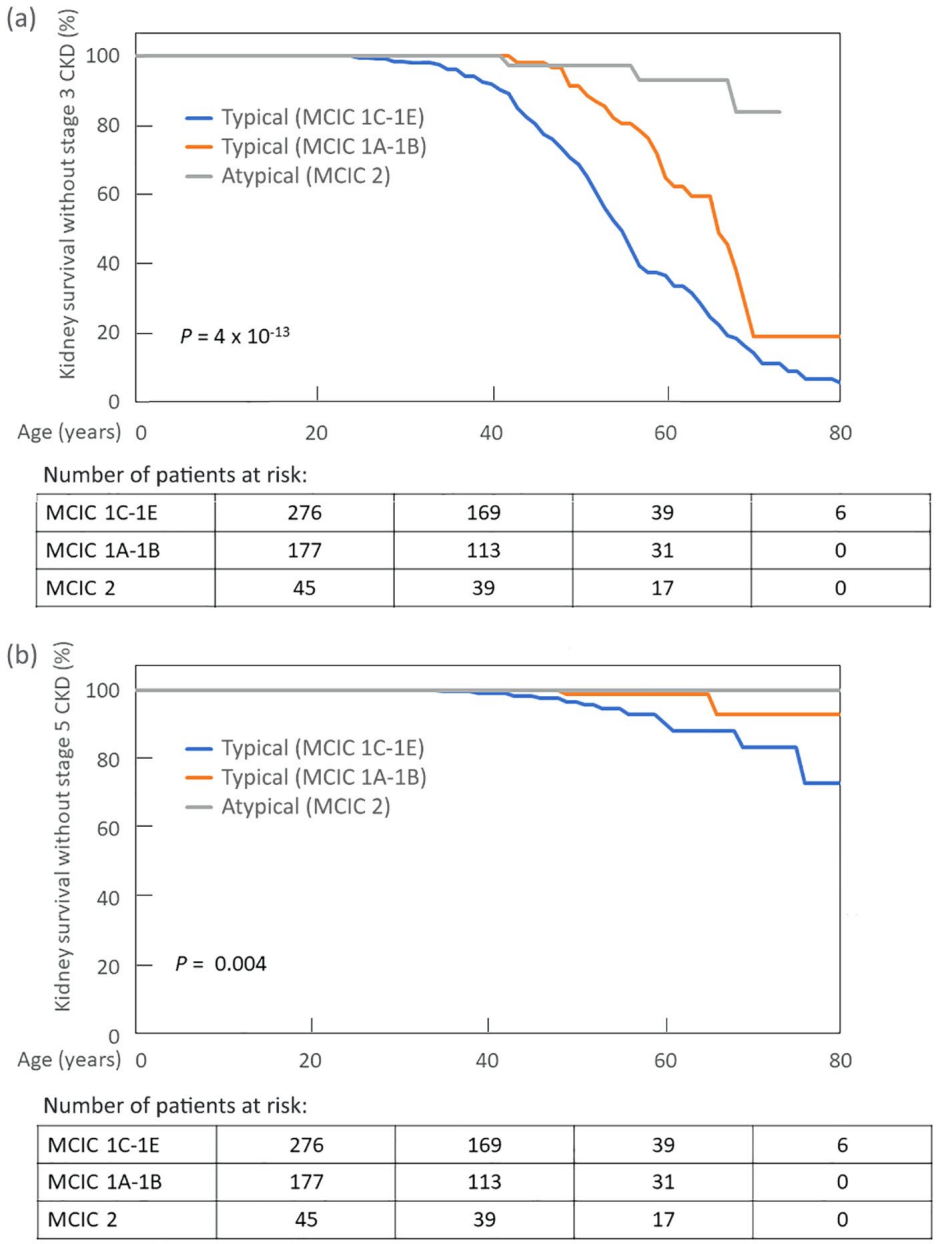
Renal survival curves in patients with atypical versus typical polycystic kidney disease by imaging. Patients with atypical polycystic kidney disease by imaging are less likely to progress to late stages of chronic kidney disease (CKD). Kaplan–Meier kidney survival (defined as the absence of CKD stage 3 or stage 5) for patients with typical Mayo Clinic Imaging Class (MCIC 1C-1E, MCIC 1A-1B) vs. atypical (MCIC 2) imaging patterns was compared using the log-rank test. Only one patient out of the 45 with MCIC 2 belonged to class MCIC 2B.

The clinical characteristics of patients with typical versus atypical kidney imaging patterns are detailed in Table 2. Patients with atypical polycystic kidney disease by imaging were older (55 versus 43 years, *P* < 0.001) with a male predominance (63.0% vs. 43.8%, *P* = 0.01); they were less likely to have a family history of ADPKD (26.1% vs. 74.6%, *P* < 0.001) or a detectable *PKD1* or *PKD2* mutation (9.2% vs. 80.4%, *P* < 0.001; Fig. 2a). Despite being more than 10 years older on average, patients with atypical kidney imaging patterns did not have a significantly different eGFR compared to those with typical kidney imaging patterns (median [IQR]: 82.0 [68.8–98.5] vs. 79.0 [49.0–101.0] mL/min/1.73 m^2^; *P* = 0.3), suggesting milder CKD in the former group. Consistent with this notion, patients with atypical kidney imaging patterns showed excellent kidney survival as defined by the absence of CKD stage 3 or stage 5 (Fig. 3; *P* < 0.001 by the log-rank test).

**Table 2:** Characteristics of the study cohort.

Patient characteristics	Typical patterns	Atypical patterns
	(n = 477)	(n = 46)
Age (years)^a^	43 (33–53)	55 (45–68)
Male sex	209 (43.8)	29 (63.0)
Positive family history of ADPKD	356 (74.6)	12 (26.1)
**Mutation class**		
*PKD1* PT	180 (37.7)	1 (2.2)
*PKD1* in-frame indel	18 (3.8)	0 (0)
*PKD1* NT^b^	106 (22.2)	3 (6.5)
*PKD2*	129 (27.0)	5 (10.9)
NMD	44 (9.2)	37 (80.4)
Serum creatinine (mg/dL)^c^	1.0 (0.8–1.4)	0.9 (0.8–1.2)
eGFR (mL/min/1.73 m^2^)^c^	79.0 (49.0–101.0)	82.0 (68.8–98.5)
TKV (mL)	1192 (626–2140)	1093 (776–1570)
Ht-TKV (mL/m)	712 (370–1239)	634 (457–874)
**Mayo clinic imaging class**		N/A
1A	51 (10.7)	
1B	133 (27.9)	
1C	144 (30.2)	
1D	84 (17.6)	
1E	65 (13.6)	

Data expressed as number (%) or median (interquartile range).

eGFR, estimated glomerular filtration rate; Ht-TKV, height-adjusted total kidney volume; NMD, no mutation detected; NT, non-truncating; PT, protein-truncating; TKV, total kidney volume.

^a^At MRI/CT scan.

^b^One patient who had both a *PKD1* NT and a *PKD2* mutation is included here.

^c^At last follow-up.

### Image analyses of atypical polycystic kidney disease

Of the 46 patients with atypical polycystic kidney disease (see Supplementary Tables S1 and S2), 45 (1 unilateral disease, 10 asymmetric disease, 29 lopsided disease, and 5 polycystic kidney disease with segmental sparing) displayed imaging patterns associated with an apparent sparing of cystic disease in one or more parts of the kidneys, which is highly unusual in patients older than 40 years of age. Notably, only 20% (9/45) of these patients had a detectable *PKD1* (n = 3 for non-truncating and n = 1 for truncating) or *PKD2* (n = 5) mutation. Only one patient in this cohort displayed the pattern of acquired unilateral atrophy, without an identifiable *PKD1* or *PKD2* mutation.

Failure to exclude patients with atypical kidney imaging patterns can lead to erroneous CKD risk prediction by the Mayo Clinic Imaging Classification. If unrecognized, 41.3% (19/46) of our patients with atypical kidney imaging patterns would have been misclassified as being at high risk for progression to ESKD (i.e., Mayo Clinic Imaging Class 1C-1E; see Fig. 2b).

Significant variability in cystic liver disease severity was observed in patients with atypical polycystic kidney disease by imaging: 29 (63.0%) had fewer than two liver cysts, 11 (23.9%) had between two and ten cysts, and 6 (13.0%) had more than ten cysts. Mild liver disease (≤ 10 cysts) was more frequent in patients with atypical imaging patterns compared to patients with typical imaging patterns (87.0% versus 46.1%, *P* < 0.001).

## Discussion

In this large cohort study from a single geographic region, we documented a prevalence of atypical polycystic kidney disease by imaging of 8.8% (46/543) which is very similar to that reported in the discovery cohort used for the Mayo Clinic Imaging Classification^10^. In the original report, patients with atypical patterns were excluded from subsequent analyses. Compared to patients with typical imaging patterns, our patients with atypical imaging patterns were older, and less likely to have a family history of ADPKD, a detectable *PKD1* or *PKD2* mutation, or progression to CKD stage 3 or stage 5. Thus, patients with atypical polycystic kidney disease by imaging have an excellent prognosis with a very low risk for ESKD. As shown in Fig. 2b, failure to appropriately identify patients with atypical patterns and incorrect Mayo Clinic Imaging Classification can lead to erroneous stratification of CKD risk. Indeed, if unrecognized as atypical, 41% of our patients with atypical kidney imaging patterns would have been misclassified as being at high risk for progression to ESKD (i.e., Mayo Clinic Imaging Class 1C–1E). Misclassification could result in implementing disease-modifying therapy in a group of patients not originally selected for the tolvaptan trials. The encouraging prognosis of patients with atypical patterns by imaging makes them unsuitable for disease-modifying therapy, with a benefit-to-risk ratio that is likely unfavourable.

To fully capture all observed atypical kidney imaging patterns, we added two additional patterns to the Mayo Clinic Imaging Classification. Firstly, a "segmental sparing” pattern, characterized by bilateral and diffuse cystic disease with sparing of one pole of one or both kidneys, which is relatively common (i.e., 5/46 or 10.9%) in this study. Secondly, a “mild lopsided” pattern, where 15–49% of TKV is attributable to 2–5 cysts (similar to the “lopsided” pattern where ≥ 50% of TKV is attributable to 2–5 cysts); it is the most common atypical imaging pattern in our cohort (i.e., 20/46 or 43.5%). Both of these patterns were strongly associated with the clinical and genetic features of atypical polycystic kidney disease, namely low probability of a positive family history, low probability of a detectable mutation, or slowly progressive kidney disease.

Forty-five of the 46 patients with atypical polycystic kidney disease (1 unilateral, 10 asymmetrical, 29 lopsided, and 5 segmental sparing) showed complete sparing of cystic disease with normal parenchyma in one or more parts of the kidneys. Sparing of more than one region of the kidney is a common finding (40/46 or 87.0% in this cohort) and is suspicious of somatic mosaicism^14,15^. Mosaicism refers to the occurrence of two genetically distinct cell populations within an individual, due to the somatic mutation of a single pluripotent stem cell during embryogenesis or development^16,17^. Due to dilution and variable involvement of the affected cells, a mosaic individual with ADPKD often presents with de novo cystic kidney disease with atypical imaging (i.e. focal, unilateral, or asymmetrical) patterns, as exemplified by the proband of TOR135 we previously reported^18^. However, the diagnosis of mosaicism is technically challenging and frequently missed by Sanger sequencing, which is frequently used for ADPKD, due to dilution of the mutation signal from the admixture of normal and mutant cells—a difficulty that can be overcome by next-generation sequencing with high-read depth^19^. Indeed, by using next-generation sequencing, a recent study of 387 *PKD1* and *PKD2* mutation-negative patients with ADPKD identified 20 *PKD1* somatic mosaics, with at least 6 of them displaying atypical kidney imaging patterns^15^. In addition to somatic mosaicism, *PKD2* (n = 5) and *PKD1* non-truncating (n = 3) mutations, which are typically associated with mild cystic kidney disease, were found in 8 of 9 (88.9%) mutation-positive patients with atypical imaging patterns in our study. Next-generation sequencing for mutation screening of additional genes, such as *PRKCSH*, *GANAB*, *ALG8*, *ALG9*, *SEC61B*, *SEC63, DNAJB11*, and *HNF1B*, which may be associated with mild or atypical polycystic kidney disease, may allow for further elucidation of the genetic causes of atypical polycystic kidney disease by imaging^14^.

In conclusion, approximately 9% of a large cohort of patients with ADPKD displayed atypical polycystic kidney disease by imaging. Compared to patients with typical kidney imaging patterns, they were older, and less likely to have a family history of ADPKD, a detectable mutation in *PKD1* and *PKD2*, or progression to advanced CKD. The causes of atypical polycystic kidney disease by imaging are heterogeneous and may include somatic mosaicism, mild disease associated with *PKD1* non-truncating or *PKD2* mutations, as well as mutations in other cystic disease genes. Incorrect identification of atypical imaging patterns can lead to inappropriate risk stratification. Elucidating the genetic causes of atypical polycystic kidney disease by imaging with next-generation sequencing, including broad cystic gene panels, will advance our understanding of this clinical syndrome and help clinicians counsel patients on the most appropriate management strategy.

## Methods

### Patient selection

Study patients (n = 543) were recruited from the extended Toronto Genetic Epidemiology Study of PKD (eTGESP), which enrolled 521 patients seen at the Centre for Innovative Management of Polycystic Kidney Disease (https://www.cimpkd.ca/) and 22 patients seen at St. Joseph’s Healthcare in Hamilton, between March 1, 2016 and September 30, 2018. All study patients fulfilled the ultrasound or magnetic resonance imaging (MRI) based diagnostic criteria for ADPKD; none had an eGFR less than 15 mL/min/1.73 m^2^ at the time of recruitment^20,21^. They were referred by more than 100 academic and community nephrologists in the Greater Toronto Area for risk stratification by kidney MRI and genetic testing, and potentially, novel therapeutic interventions. All study patients provided informed consent to a pre-specified research protocol approved by the Research Ethics Board at the University Health Network in Toronto and St. Joseph’s Healthcare in Hamilton, both in Ontario, Canada. Research was performed in accordance with the Declaration of Helsinki.

### Exposure and outcomes

All study patients completed a standardized clinical questionnaire that included their demographics, detailed family history (with an annotated pedigree available for every proband in the study), and potential complications of ADPKD. In addition, their serum creatinine, *PKD1* and *PKD2* mutation results, and MRI or computed tomography (CT) images were collected and used for the analyses. Their estimated glomerular filtration rate (eGFR) was calculated using the Chronic Kidney Disease Epidemiology Collaboration (CKD-EPI) equation from the last clinic follow-up^22^.

TKV was assessed by an experienced radiologist (MP) using the ellipsoid formula (4πabc/3, where a, b, and c are the orthogonal semi-axis lengths). All images were visually inspected for polycystic kidneys with atypical imaging patterns as outlined in the Mayo Clinic Imaging Classification^10^. Two additional categories (segmental sparing and mild lopsided) were included in our classification to cover atypical presentations not accounted for in the original paper; they were seen relatively frequently in our clinical experience to warrant their inclusion in this study. Segmental sparing is defined by generalized cystic disease with sparing of one pole of one or both kidneys. Mild lopsided was introduced to describe patterns suggestive of lopsided but with dominant cysts amounting to 15–49%, as opposed to equal to or greater than 50%, of TKV. A systematic analysis of cyst patterns was conducted on MRI or CT scan independently by two different investigators (IAI and AZW), including one radiologist (AZW). Whenever the investigators differed as to the interpretation and could not come to an agreement, a senior abdominal radiologist (KK) was brought in to settle ambiguous cases.

Mutation screening for *PKD1* and *PKD2* was performed by targeted exome sequencing as per the published protocol^23^. All pathogenic mutations identified through targeted exome sequencing were confirmed by Sanger sequencing using a validated PCR protocol^18^. All nonsense, frameshift, and canonical splice-site mutations were grouped as protein-truncating mutations, and non-synonymous missense or atypical splice site mutations were grouped as non-truncating mutations. In-frame insertions/deletions (in-frame indel) were classified separately. Non-truncating mutations were evaluated for their pathogenicity using bioinformatics prediction algorithms (Align GVGD, PolyPhen-2, SIFT, PROVEAN, and Human Splicing Finder), review of the PKD mutation database (http://pkdb.mayo.edu), and evaluation of familial co-segregation whenever possible^18^. All mutation-negative patients were re-screened by multiplex ligation-dependent probe amplification to detect large gene rearrangements^24^.

### Statistical analysis

Biochemical, genetic, and volumetric parameters were compared between the participants with polycystic kidneys and typical or atypical imaging patterns. Statistical analysis was performed in GraphPad Prism and R. Categorical variables were reported as frequency (percentage) and normally distributed continuous variables were reported as mean ± standard deviation, while non-normal continuous variables were reported as median (interquartile range, IQR). Patient characteristics were compared using the Mann–Whitney test and Fisher’s exact test. Kaplan–Meier curves were plotted to compare kidney survival (defined as the absence of CKD stage 3 and stage 5, respectively) for patients with typical versus atypical polycystic kidney disease by imaging, and tested for statistical significance using the log-rank test. Censoring was done at death, development of CKD stage 3 or 5, or age at the most recent follow-up.

## Supplementary Information

Supplementary Information.

## Data Availability

The datasets generated and analyzed during the current study are available from the corresponding author on reasonable request.
