# Supplementary material for: Atypical Polycystic Kidney Disease as defined by Imaging
Source: Sci Rep. 2023 Feb 20;13:2952. doi: 10.1038/s41598-022-24104-w (PMC9941465; doi:10.1038/s41598-022-24104-w)
Supplement: Supplementary file 1 — Supplementary Information. [file 41598_2022_24104_MOESM1_ESM.docx]

**Supplementary Materials**

**Supplementary Table 1. Individual characteristics of patients with atypical imaging patterns**

| **Atypical imaging pattern** | **Age (years)^a^** | **Sex** | **Family history of ADPKD** | **Mutation class** | **eGFR**  **(mL/min/1.73m^2^)^b^** | **TKV (mL)** | **Potential**  **misclassification**  **with MCIC** | **Liver cyst count** |
| --- | --- | --- | --- | --- | --- | --- | --- | --- |
| Unilateral | 53 | M | Yes | NMD | 101 | 582 | 1A | >10 |
| Asymmetric | 42 | F | Yes | *PKD1* NT | 102 | 615 | 1B | >10 |
| Asymmetric | 19 | F | No | *PKD1* NT | 110 | 788 | 1E | 0 |
| Asymmetric | 62 | M | Yes | NMD | 71 | 1306 | 1B | 0 |
| Asymmetric | 64 | M | No | NMD | 91 | 1147 | 1B | 0 |
| Asymmetric | 40 | M | No | NMD | 96 | 437 | 1A | 0 |
| Asymmetric | 42 | F | No | NMD | 101 | 782 | 1B | >10 |
| Asymmetric | 74 | M | No | NMD | 46 | 2763 | 1C | 2 |
| Asymmetric | 48 | M | No | NMD | 94 | 1204 | 1C | 2 |
| Asymmetric | 38 | F | No | NMD | 112 | 659 | 1B | 0 |
| Asymmetric | 34 | F | No | NMD | 78 | 823 | 1C | 0 |
| Lopsided | 53 | F | Yes | *PKD2* | 82 | 701 | 1B | >10 |
| Lopsided | 54 | M | Yes | NMD | 62 | 1208 | 1B | 0 |
| Lopsided | 55 | M | Yes | NMD | 68 | 3632 | 1D | 1 |
| Lopsided | 59 | M | No | NMD | 116 | 2274 | 1C | 1 |
| Lopsided | 58 | F | No | NMD | 50 | 781 | 1B | 0 |
| Lopsided | 68 | M | No | NMD | 79 | 1031 | 1B | 3 |
| Lopsided | 57 | M | No | NMD | 80 | 2082 | 1C | 0 |
| Lopsided | 72 | M | No | NMD | 86 | 1713 | 1B | 3 |
| Lopsided | 57 | M | No | NMD | 71 | 1929 | 1C | 0 |
| Acquired unilateral atrophy | 69 | M | No | NMD | 107 | 1269 | 1B | 3 |
| Segmental sparing | 33 | F | No | *PKD1* NT | 69 | 1014 | 1C | >10 |
| Segmental sparing | 41 | F | Yes | *PKD2* | 28 | 1912 | 1D | 0 |
| Segmental sparing | 49 | M | Yes | NMD | 61 | 1121 | 1B | 0 |
| Segmental sparing | 34 | M | No | NMD | 102 | 779 | 1C | 1 |
| Segmental sparing | 70 | M | No | NMD | 86 | 2580 | 1C | 4 |
| Mild lopsided | 60 | M | No | *PKD1* PT | 81 | 1554 | 1C | 4 |
| Mild lopsided | 69 | M | Yes | *PKD2* | 60 | 767 | 1B | 5 |
| Mild lopsided | 51 | F | Yes | *PKD2* | 63 | 1728 | 1C | 4 |
| Mild lopsided | 38 | F | Yes | *PKD2* | 104 | 1970 | 1D | 0 |
| Mild lopsided | 50 | F | Yes | NMD | 62 | 623 | 1B | 0 |
| Mild lopsided | 62 | M | No | NMD | 61 | 1273 | 1B | 0 |
| Mild lopsided | 47 | M | No | NMD | 87 | 682 | 1B | 0 |
| Mild lopsided | 68 | M | No | NMD | 77 | 1085 | 1B | 3 |
| Mild lopsided | 73 | M | No | NMD | 103 | 1095 | 1B | 0 |
| Mild lopsided | 68 | M | No | NMD | 89 | 1332 | 1B | 1 |
| Mild lopsided | 74 | M | No | NMD | 91 | 844 | 1B | 0 |
| Mild lopsided | 40 | F | No | NMD | 80 | 1210 | 1C | 1 |
| Mild lopsided | 67 | M | No | NMD | 73 | 674 | 1A | 0 |
| Mild lopsided | 44 | F | No | NMD | 74 | 1451 | 1C | 0 |
| Mild lopsided | 45 | F | No | NMD | 98 | 1090 | 1C | 0 |
| Mild lopsided | 74 | M | No | NMD | 87 | 822 | 1B | 0 |
| Mild lopsided | 54 | F | No | NMD | 100 | 752 | 1B | >10 |
| Mild lopsided | 61 | M | No | NMD | 82 | 1626 | 1C | 0 |
| Mild lopsided | 53 | M | No | NMD | 55 | 1079 | 1B | 10 |
| Mild lopsided | 69 | F | No | NMD | 93 | 480 | 1A | 0 |

Abbreviations: eGFR, estimated glomerular filtration rate; MCIC, Mayo Class Imaging Classification; NMD, no mutation detected; NT, non-truncating; PT, protein-truncating; TKV, total kidney volume; ^a^age at TKV measurement; ^b^at last follow-up.

**Supplementary Table 2. Characteristics of patients by atypical category**

| Category |  | n | Mean age | Positive family history (%) | Mutation class | Mean serum creatinine (mg/dL) | Mean eGFR (mL/min/1.73m^2^) | Median TKV (mL) |
| --- | --- | --- | --- | --- | --- | --- | --- | --- |
| 2A | Unilateral | 1 | 53 | 100% | NMD | 0.59 | 101.0 | 582 |
|  | Segmental | 0 |  |  |  |  |  |  |
|  | Asymmetric | 10 | 46 | 20% | NMD (8), *PKD1* NT (2) | 0.92 | 90.0 | 806 |
|  | Lopsided | 9 | 59 | 33% | NMD (8), *PKD2* (1) | 1.05 | 77.1 | 1713 |
| 2B | Acquired unilateral atrophy | 1 | 69 | 0% | NMD | 0.64 | 107.0 | 1269 |
|  | Acquired bilateral atrophy | 0 |  |  |  |  |  |  |
| Additional | Segmental sparing | 5 | 45 | 40% | NMD (3), *PKD1* NT (1), *PKD2* (1) | 1.25 | 69.2 | 1121 |
|  | Mild lopsided | 20 | 58 | 20% | NMD (16), *PKD1* PT (1), *PKD2* (3) | 0.95 | 81.0 | 1088 |

Abbreviations: eGFR, estimated glomerular filtration rate; NMD, no mutation detected; NT, non-truncating; PT, protein-truncating; TKV, total kidney volume.

**Supplementary Figure 1**


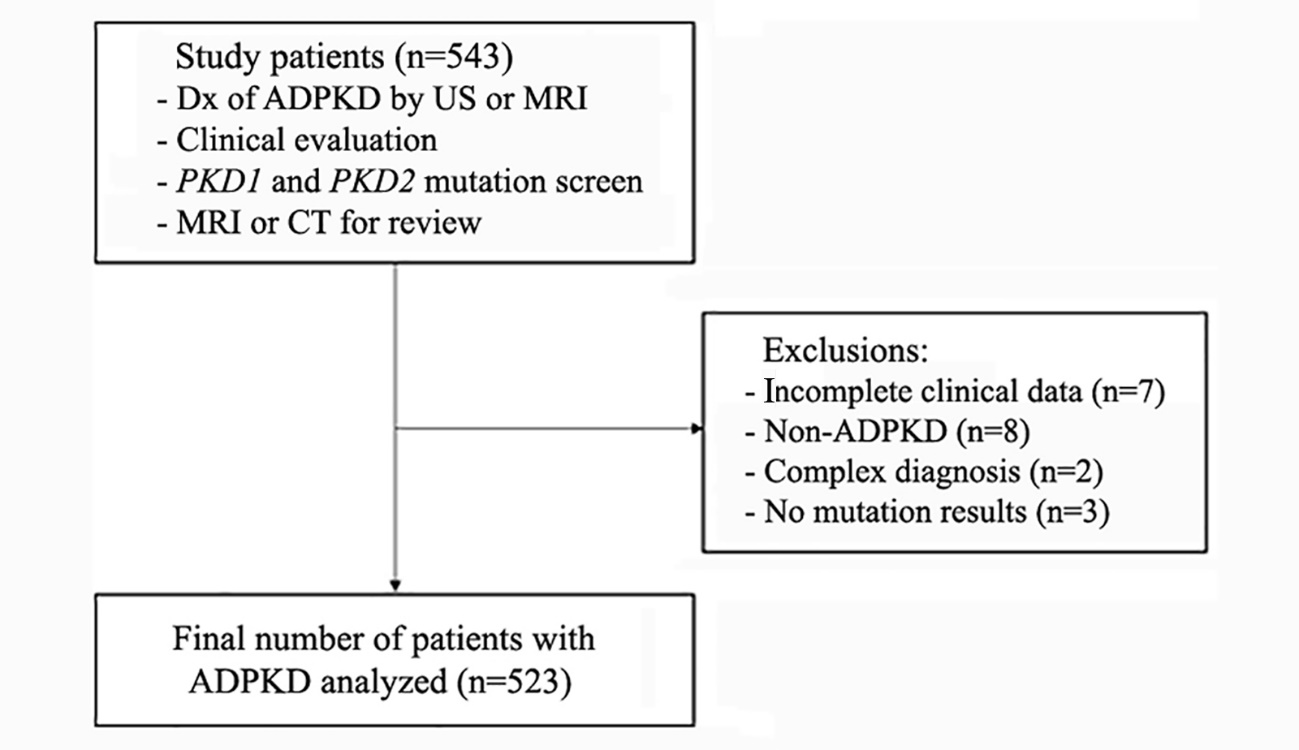


**Study flow chart.** A total of 523 patients were analyzed from a cohort of 543 patients with presumed ADPKD who were initially evaluated in this study. Twenty cases were excluded because of: incomplete clinical data (n=7); non-ADPKD diagnoses (n=8) including simple cysts (n=4), peri-pelvic cysts (n=2), congenital anomalies of the kidney and urinary tract (n=1), and cystic disease related to a *COL4A1* mutation (n=1); complex diagnoses of ADPKD with a second kidney disease (n=2); and no *PKD1* and *PKD2* mutation results available (n=3).
